# Block-Segmented Spatial-Spectrum Imaging for Near-Field Millimeter Waves with Microwave-Photonic Broadband Reception

**DOI:** 10.3390/s26216796

**Published:** 2026-10-27

**Authors:** Yang Zhao, Zhiting Li, Baode Lin, Xing Li, Weiwen Zou

**Affiliations:** 1 State Key Laboratory of Photonics and Communications, Intelligent Microwave Lightwave Integration Innovation Center, Department of Electronic Engineering, Shanghai Jiao Tong University, Shanghai 200240, China; zhaoyang20@sjtu.edu.cn (Y.Z.); lzt3391@126.com (Z.L.); linbd2023@sjtu.edu.cn (B.L.); wzou@sjtu.edu.cn (W.Z.); 2 State Key Laboratory of Micro-Nano Engineering Science, Intelligent Microwave Lightwave Integration Innovation Center, Department of Electronic Engineering, Shanghai Jiao Tong University, Shanghai 200240, China

**Keywords:** millimeter-wave imaging, near-field radar, microwave photonics, security screening, spatial-spectrum imaging

## Abstract

**Highlights:**

**What are the main findings?**

- A block-segmented spatial-spectrum reconstruction method converts wide-area near-field millimeter-wave imaging into multiple locally referenced spectral reconstruction subproblems, thereby suppressing nonlinear phase errors caused by the breakdown of the global far-field approximation.
- A Ka-band microwave-photonic broadband reception prototype operating over 32–38 GHz experimentally achieves centimeter-level imaging resolution of approximately 3.3 cm × 4.7 cm and reconstructs a gun-shaped threat analog using an emulated 3-transmit/13-receive aperture.

**What are the implications of the main findings?**

- The proposed method extends the effective near-field imaging region from a locally focused 0.2 m × 0.2 m area to a 1 m × 1 m imaging plane, corresponding to a fivefold increase in linear span and a 25-fold increase in imaging area.
- By combining FFT-compatible block-segmented spatial-spectrum processing with microwave-photonic broadband reception, the approach provides a feasible technical route toward compact, wide-area, high-resolution, and computationally efficient millimeter-wave security-screening systems.

**Abstract:**

Active millimeter-wave (mm-wave) imaging uses non-ionizing electromagnetic radiation to detect concealed metallic and dielectric objects and is an important technology for personnel security screening. However, practical security-screening scenarios typically involve short standoff distances and small apertures: the limited physical aperture constrains the two-dimensional imaging resolution, while the short antenna-to-target distance introduces non-negligible high-order nonlinear phase errors for scatterers far from the global coordinate origin, leading to image defocusing. This paper presents a block-segmented spatial-spectrum near-field imaging method combined with a Ka-band microwave-photonic broadband reception prototype. Microwave-photonic broadband reception provides broadband spectral resources to expand the two-dimensional spatial-frequency support, thereby improving imaging resolution under limited-aperture conditions. Meanwhile, the full imaging plane is divided into multiple subblocks, each of which establishes its own local coordinate system. Scattering points within each subblock are kept close to the reference origin to suppress high-order nonlinear phase errors, thereby enhancing the accuracy of local linear spatial-frequency mapping. Finally, global near-field imaging is achieved through sub-image stitching. Simulations with point targets and an aircraft-shaped extended target show that, compared with spatial-spectrum imaging based on a single global coordinate origin, the proposed method effectively suppresses nonlinear phase errors and enables large-area near-field imaging. A discrete-component laboratory prototype implements microwave-photonic broadband reception for a time-division 3-transmit/13-receive acquisition configuration operating from 32 to 38 GHz. The method extends the effective linear imaging span from a 0.2 m × 0.2 m locally focused region to a 1 m × 1 m imaging plane, corresponding to a fivefold increase in linear span and a 25-fold increase in area. Based on the antenna arrangement geometry and the 32–38 GHz operating frequency band, the theoretical two-dimensional imaging resolution of the prototype system is estimated to be approximately 3 cm × 4 cm. Experiments using aluminum-foil strip resolution targets demonstrate that the prototype can resolve adjacent strip scattering regions spaced approximately 3.3 cm and 4.7 cm apart in two orthogonal directions, with a corresponding measured imaging resolution of approximately 3.3 cm × 4.7 cm. The prototype is also capable of reconstructing the primary scattering structure of gun-shaped threat simulation targets. These results indicate that combining block-segmented spatial-spectrum imaging with microwave-photonic broadband reception provides a potentially feasible approach for high-resolution, wide-area millimeter-wave security-screening imaging.

## 1. Introduction

### 1.1. Background and Literature Review

Millimeter-wave security imaging has become an important electromagnetic sensing technology for airports, transportation hubs, and other public inspection scenarios. Compared with X-ray screening, mm-wave illumination is non-ionizing; and compared with optical imaging, it can penetrate common clothing, packaging materials, and many non-metallic coverings. Therefore, mm-wave imaging can provide contactless inspection while balancing penetration capability, exposure safety, and privacy protection [1,2,3,4]. In actual security-screening systems, the antenna is typically located relatively close to the target. As the target’s scattering points gradually deviate from the origin of the global Cartesian coordinate system, the far-field imaging approximation gradually fails. Achieving wide-area, high-speed near-field imaging while ensuring high-resolution imaging of threatening targets has become a key challenge facing millimeter-wave security-screening systems.

To meet these requirements, existing studies have enhanced mm-wave near-field imaging mainly through array design, aperture sampling, and reconstruction algorithms. Large-scale multiple-input multiple-output (MIMO) arrays and synthetic-aperture configurations increase spatial diversity and effective aperture size, thereby improving image resolution and target-detail representation [2,5]. Sparse-array and learning-assisted methods reduce the number of physical observation channels and the acquisition burden, but their robustness depends on accurate calibration, representative training data, and stable deployment conditions [6,7]. Regardless of whether dense or sparse apertures are used, the ability to rapidly and accurately reconstruct multichannel near-field echoes remains a decisive factor in system-level imaging performance. Backprojection (BP) and generalized synthetic-aperture focusing techniques (GSAFT) can accurately model spherical propagation and accommodate flexible acquisition geometries, but their coherent summation over every pixel or voxel becomes computationally expensive for high-resolution and wide-field screening [3,5,8,9]. To improve computational efficiency, the range migration algorithm (RMA), wavenumber-domain imaging, frequency-domain scaling, nonuniform fast Fourier transform (NUFFT), and FiNUFFT-based methods have been developed to replace exhaustive pixel-wise accumulation with spectral-domain mapping and fast Fourier processing [10,11,12,13,14].

### 1.2. Research Gap

Spatial-spectrum imaging is a fast frequency-domain reconstruction method that maps multichannel echoes onto a spatial-frequency matrix and uses the fast Fourier transform (FFT) for efficient image inversion. Under far-field conditions, broadband frequency resources can expand spatial-frequency coverage and work together with aperture-based spatial sampling to improve azimuth and cross-range resolution [3,10,12]. However, conventional global spatial-spectrum imaging typically relies on a single far-field or local plane-wave approximation to establish an approximately stable Fourier duality between target reflectivity and spatial frequency. In near-field security-screening scenes, the transmit and receive propagation geometries vary significantly for scatterers at different spatial positions, making a unified global approximation difficult to satisfy. When a spatial spectrum is constructed with respect to one global reference origin, high-order phase terms become non-negligible in regions far from that origin, causing phase-map mismatch, spatial-frequency misregistration, and image defocus. In parallel, microwave-photonic radar and imaging architectures provide broad instantaneous bandwidth, low-loss distribution, flexible waveform generation, and strong immunity to electromagnetic interference, offering a hardware basis for extending spatial-frequency support and improving mm-wave imaging resolution [15,16,17,18]. Against this background, the key problem is how to exploit broadband spectral resources while suppressing near-field defocusing caused by nonlinear propagation phase errors.

The unresolved challenge is to maintain accurate focusing across an extended near-field imaging plane while fully exploiting broadband spatial-frequency support without incurring the pixel-wise computational cost of exact spherical-wave reconstruction. In particular, increased RF bandwidth alone cannot correct spatially varying phase errors, whereas a single global linearization does not remain valid over an electrically large target region.

### 1.3. Novelty of the Proposed Method

This study decomposes the wide-area near-field imaging problem into multiple local spatial-spectrum reconstruction problems to address the phase mismatch in global spatial-spectrum imaging under near-field conditions. The strategy of using multiple local references is conceptually similar to multiple-reference approaches in extended-object modeling. These approaches employ multiple reference models to estimate an extended object’s kinematic state and shape, whereas the local references in this study are used for propagation phase compensation and spatial-frequency mapping [19]. Thus, this study combines phase compensation based on local coordinates with near-field spatial-spectrum imaging to reduce higher-order propagation phase errors and extend the effective imaging area. The proposed method first partitions the imaging plane into subblocks and assigns an independent local coordinate origin to each subblock. The corresponding transmit and receive propagation phases are then compensated, followed by local spatial-spectrum construction and image inversion for each subblock. Finally, the local sub-images are transformed back to the global coordinate system and stitched into the complete image. By introducing local coordinate reconstruction, the proposed approach reduces the influence of high-order phase terms within each subregion, thereby suppressing nonlinear near-field phase errors while retaining the low computational complexity of FFT-compatible spatial-spectrum processing. Simulation and experimental results show that, compared with spatial-spectrum imaging based on a single global coordinate origin, the proposed method suppresses near-field defocusing and expands the effective imaging area by a factor of 25. Combined with microwave-photonic broadband reception, the broader spectral resources further extend the spatial-frequency coverage, thereby alleviating the resolution limitations imposed by small-aperture configurations in practical security-screening scenarios.

The methodological novelty is not the partitioning of an image after reconstruction, but the use of subblock-specific transmit–receive phase compensation and spatial-frequency mapping before inverse Fourier reconstruction. Each subblock reuses the same acquired multistatic echo dataset, so the approach does not require separate measurements or antenna assignments for different target regions. This local Fourier-domain treatment is integrated with broadband microwave-photonic reception to combine extended near-field focusing with two-dimensional spatial-frequency coverage under a limited aperture.

### 1.4. Main Contributions

The main contributions of this paper are summarized as follows:

1. A block-segmented spatial-spectrum imaging model is formulated for two-dimensional near-field mm-wave imaging.
2. The relationship among local coordinate selection, residual nonlinear phase, and image defocus is clarified from an implementation-oriented radar signal-processing perspective.
3. Simulations verify that segmentation extends the effective imaging field from a local 0.2 m × 0.2 m well-focused region to a full 1 m × 1 m imaging plane for point-target scenes and improves the reconstruction of an aircraft-shaped extended target.
4. A Ka-band near-field imaging experimental system is constructed, in which a microwave-photonic broadband receiver acquires broadband echoes from an emulated 3-transmit/13-receive aperture over 32–38 GHz. Broadband spatial-spectrum resolution and effective imaging of a gun-shaped threat analog under wide-area near-field conditions are experimentally verified.

## 2. Models and Methods

### 2.1. Block-Segmented Spatial-Spectrum Imaging Model

In a Cartesian imaging coordinate system, the target scene is modeled as a two-dimensional reflectivity distribution. The proposed reconstruction method estimates this distribution from the phase history of the received wideband echoes. The echo signal model can be expressed as

(1) $$s\left(t\right)=\sum \sigma \left(x_{0},y_{0}\right)e^{j2\mathrm{\pi f}\left(t-\frac{r_{T}+r_{R}}{c}\right)}$$

Here, *r_T_* and *r_R_* denote the propagation distances from the transmitting and receiving antennas to a target scattering point, respectively, and *σ*(*x*_0_,*y*_0_) denotes the scattering-point distribution of the target. The amplitude term is temporarily neglected because the defocus considered in this study is dominated by the phase error caused by the extension of the imaging field. The propagation distances in the phase term are given by

(2) $$r_{T}=\sqrt{{\left(x_{T}-x_{o}\right)}^{2}+{\left(y_{T}-y_{o}\right)}^{2}}$$

(3) $$r_{R}=\sqrt{{\left(x_{R}-x_{o}\right)}^{2}+{\left(y_{R}-y_{o}\right)}^{2}}$$

where (*x_T_*,*y_T_*) and (*x_R_*,*y_R_*) are the positions of the transmitting and receiving antennas in the established Cartesian coordinate system, respectively, and (*x*_0_,*y*_0_) denotes the coordinates of a target scattering point. After Fourier transformation and removing the transmitted waveform in the frequency domain, the received data can be written as

(4) $$S\left(f\right)=\sum \sigma \left(x_{0},y_{0}\right)e^{j2\mathrm{\pi f}\left(\frac{r_{T}+r_{R}}{c}\right)}$$

The propagation distance in the phase term can be approximated by a Taylor expansion around the origin of the selected imaging coordinate system. Retaining the first-order terms produces a linear relationship between the target coordinates and spatial frequency

(5) $$r_{T}=r_{1}-\frac{x_{T}}{r_{1}}x_{0}-\frac{y_{T}}{r_{1}}y_{0}+\frac{1}{2}\left(\frac{y_{T}^{2}}{{\left(x_{T}^{2}+y_{T}^{2}\right)}^{3/2}}{x_{0}}^{2}-2\frac{y_{T}x_{T}}{{\left(x_{T}^{2}+y_{T}^{2}\right)}^{3/2}}x_{0}y_{0}+\frac{x_{T}^{2}}{{\left(x_{T}^{2}+y_{T}^{2}\right)}^{3/2}}{y_{0}}^{2}\right)$$

(6) $$r_{R}=r_{2}-\frac{x_{R}}{r_{2}}x_{0}-\frac{y_{R}}{r_{2}}y_{0}+\frac{1}{2}\left(\frac{y_{R}^{2}}{{\left(x_{R}^{2}+y_{R}^{2}\right)}^{3/2}}{x_{0}}^{2}-2\frac{y_{R}x_{R}}{{\left(x_{R}^{2}+y_{R}^{2}\right)}^{3/2}}x_{0}y_{0}+\frac{x_{R}^{2}}{{\left(x_{R}^{2}+y_{R}^{2}\right)}^{3/2}}{y_{0}}^{2}\right)$$

where *r*_1_ and *r*_2_ are the distances of the transmit and receive antennas, respectively, from the origin of the coordinate system. When the distance from the antenna to the origin is much greater than the distance from the target scattering point to the origin, the higher-order terms are very small compared to the first-order terms and can be neglected. This approximation is therefore valid only within a limited local region. Assuming the far-field approximation holds, the echo is expressed as

(7) $$S\left(f\right)=\sum \sigma \left(x_{0},y_{0}\right)e^{-j2\mathrm{\pi f}\left(\frac{r_{1}+r_{2}}{c}+\frac{x_{T}}{r_{1}}x_{0}-\frac{y_{T}}{r_{1}}y_{0}+\frac{x_{R}}{r_{2}}x_{0}-\frac{y_{R}}{r_{2}}y_{0}\right)}$$

The distances from the transmit and receive antennas to the origin of the imaging coordinate system are known. Next, the constant terms in the phase expression corresponding to the distances from the transmit and receive antennas to the origin must be corrected by multiplying them by the exponential term *exp*(−*j*2*πf*(*r*_1_ *+ r*_2_)/*c*), so that the final spatial phase variation depends solely on the target’s coordinate position. The formula after phase correction is

(8) $$S\left(f\right)=\sum \sigma \left(x_{0},y_{0}\right)e^{-j2\mathrm{\pi f}\left(\frac{x_{T}}{r_{1}}x_{0}-\frac{y_{T}}{r_{1}}y_{0}+\frac{x_{R}}{r_{2}}x_{0}-\frac{y_{R}}{r_{2}}y_{0}\right)}$$

After phase correction, the remaining first-order phase term establishes the Fourier duality between the target coordinates and spatial frequency. The measured frequency-domain samples are then mapped onto the spatial-frequency coordinates of the target projection:

(9) $$S\prime \left(f\right)=\sum \sigma \left(x_{0},y_{0}\right)e^{-j2\pi \left(k_{x}x_{0}+k_{y}y_{0}\right)}$$

where

(10) $$k_{x}=f(\mathrm{fc},B)(\frac{x_{T}}{r_{1}}+\frac{x_{R}}{r_{2}}),\;\;k_{y}=f(\mathrm{fc},B)(\frac{y_{T}}{r_{1}}+\frac{y_{R}}{r_{2}})$$

where *f_c_* is the carrier frequency and *B* is the instantaneous signal bandwidth. The above expression specifies the spatial-frequency coordinates associated with different frequency samples and antenna observation directions. Here, *k_x_* and *k_y_* denote the spatial frequencies along the *x*- and *y*-directions, respectively. The discrete spatial-frequency samples are filled into an empty two-dimensional matrix to form the spatial-spectrum matrix. After the available samples are assigned to the corresponding two-dimensional spectral grid, the target image is reconstructed by an inverse Fourier transform.

Equation (9) establishes the Fourier duality relationship between the target scattering distribution and its spatial spectrum. According to this relationship, the minimum resolvable spacing in each imaging direction is inversely proportional to the spatial-spectrum support size (spatial frequency range) in the corresponding direction. Since the spatial frequency range in each direction is defined as the spatial bandwidth, the larger the spatial bandwidth, the lower the imaging resolution. The imaging resolution *R_d=x_*_,*y*_ can be expressed as

(11) $$R_{d=x,y}=\frac{1}{k_{\max.d}-k_{\min.d}}$$

where *k_max_*_,*d*_ denotes the maximum spatial frequency in the *x*- or *y*-direction, and *k_minx,d_* denotes the minimum spatial frequency in the *x*- or *y*-direction.

The spatial frequency is determined by the carrier frequency, the signal bandwidth, and the angular coverage provided by the antenna positions; therefore, imaging resolution is also influenced by these factors. When the antenna position is fixed, the spatial frequency range can be expanded by increasing the signal bandwidth. This is also the approach taken in this paper, which uses a microwave-photonic broadband receiver to achieve broadband echo acquisition. By expanding the signal frequency range, the spatial-frequency support (spatial bandwidth) is broadened within the given antenna geometry, thereby enhancing the two-dimensional imaging resolution.

(12) $$H=\left[\begin{array}{cccc} \mathrm{index} & k_{x} & k_{y} & S^{\prime }\left(f\right)\\ 1 & k_{x,1} & k_{y,1} & {S^{\prime }}_{1}\left(f\right)\\ 2 & k_{x,2} & k_{y,2} & {S^{\prime }}_{2}\left(f\right)\\ \vdots & \vdots & \vdots & \vdots \\ N & k_{x,N} & k_{y,N} & {S^{\prime }}_{N}\left(f\right) \end{array}\right]$$

where *H* denotes the spatial-spectrum matrix, in which *N* measured samples are mapped to *N* spatial-frequency coordinates. By applying a two-dimensional inverse Fourier transform to this matrix, the spatial distribution of the target reflectivity can be reconstructed.

*Image*(*x*,*y*) = *IFFT*(*H*) (13)

where *Image*(*x*, *y*) represents the reconstructed two-dimensional scattering distribution of the target. When the far-field approximation is not satisfied, the nonlinear phase error Δ*φ* in the propagation phase can be expressed as

(14) $$∆\varphi =\sum_{i=T,R}-\frac{2\mathrm{\pi f}}{c}\left(\frac{1}{2}\left(\frac{y_{i}^{2}}{{\left(x_{i}^{2}+y_{i}^{2}\right)}^{3/2}}{x_{0}}^{2}-2\frac{y_{i}x_{i}}{{\left(x_{i}^{2}+y_{i}^{2}\right)}^{3/2}}x_{0}y_{0}+\frac{x_{i}^{2}}{{\left(x_{i}^{2}+y_{i}^{2}\right)}^{3/2}}{y_{0}}^{2}\right)\right)$$

where *i* represents either the transmitting antenna or the receiving antenna, as the process involves a round-trip interaction between transmission and reception. By incorporating the nonlinear phase error into Equation (9), we obtain

(15) $$S^{\prime }\left(f\right)=\sum \sigma \left(x_{0},y_{0}\right)e^{-j2\pi \left(k_{x}x_{0}+k_{y}y_{0}+∆\varphi \right)}$$

**Figure 1:**
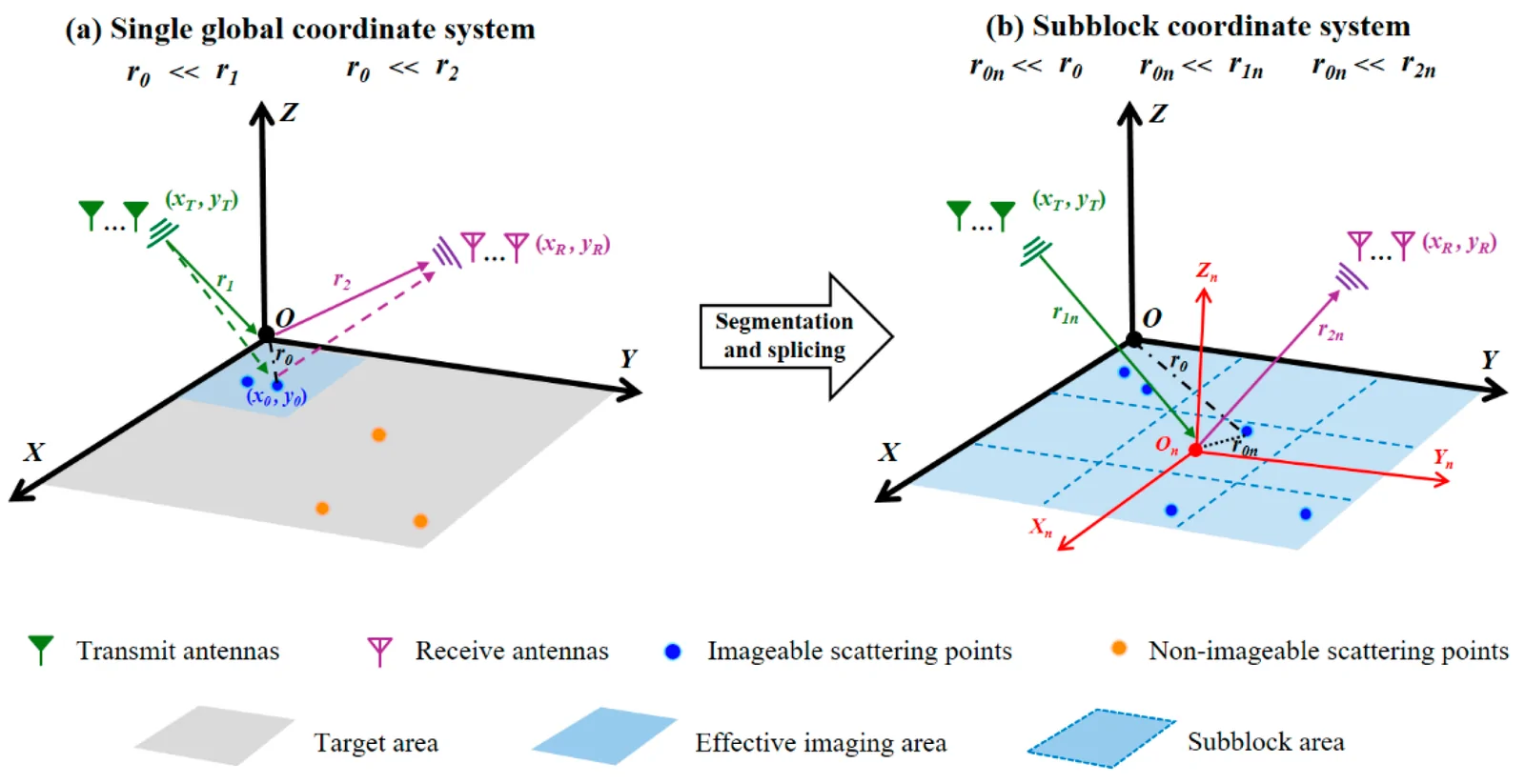
Imaging geometry and coordinate definitions. (**a**) Single global coordinate system. (**b**) Subblock coordinate system for the proposed block-segmented method. The green and purple dashed lines represent the propagation distances from the transmitting and receiving antennas to the target scattering point, respectively. The black dash-dotted line indicates the distance from the scattering point to the global coordinate origin, whereas the black dashed line indicates the distance from the scattering point to the local coordinate origin.

When the far-field approximation does not hold, nonlinear phase errors cause a mismatch between spatial frequency and spatial-frequency points in the spatial-spectrum matrix, resulting in defocused time-domain images in inverse Fourier transform inversion. As some target scattering points move farther away from the coordinate origin, the far-field approximation gradually becomes invalid, and the phase mismatch caused by higher-order propagation phase terms increases, making these scattering points difficult to focus accurately. Therefore, the effective imaging region in which the far-field approximation is satisfied and accurate focusing can be achieved is mainly confined to the vicinity of the coordinate origin. As shown in Figure 1a, only the blue scattering points within the effective imaging region are close to the global coordinate origin O and therefore satisfy the local far-field approximation, enabling effective focusing and imaging. In contrast, the scattering points are far from the global coordinate origin, and their higher-order propagation phase errors cannot be neglected.

To address this issue, the entire imaging area is divided into several subblock areas, and a separate local Cartesian coordinate system (*X_n_*, *Y_n_*, *Z_n_*) is established within each subblock area, as shown in Figure 1b. The effective imaging range is expanded from the vicinity of the global origin to the entire target area by positioning the scattering points within each subblock area close to their respective local coordinate origins. In each local coordinate system, the spatial-frequency coordinates and spectral samples are calculated independently to obtain the corresponding sub-image. Through this block-segmented strategy, every scatterer in the imaging region is associated with a nearby local origin, which reduces its spatial offset from the reference origin and suppresses high-order nonlinear phase terms in the propagation-distance expansion. This restores the validity of the local far-field approximation, enables linear spatial-frequency mapping, and allows the target to be reconstructed using the spatial-spectrum imaging model. Suppose that the full imaging region is divided into *M* × *N* subregions, and the subregion at index (*i*, *j*) is processed independently, where *i* = 1, 2, …, M and *j* = 1, 2, …, N. To ensure that the propagation phase within each subblock satisfies the local far-field approximation, the maximum subblock size D and its distance R from the antenna array should satisfy the far-field condition.

These local coordinate systems are computational reference frames introduced during image reconstruction. The measured multi-antenna echo dataset is not reacquired for different subblocks, and the subblocks are not associated with individual antennas or antenna subarrays. Instead, all available transmit–receive channels contribute to every local spatial-spectrum reconstruction, while only the phase-compensation reference origin and the spatial-frequency mapping are changed from one subblock to another.

(16) $$R>\frac{{2D}^{2}}{\lambda }$$

where *λ* is the shortest operating wavelength of the transmitted signal. This condition provides a practical design criterion for selecting the maximum subblock size. Smaller subblocks reduce the spatial offset of scatterers relative to the local coordinate origin, thereby suppressing high-order nonlinear phase errors and improving the accuracy of the local approximation. However, decreasing the subblock size increases the number of local reconstructions and the associated computational load. Therefore, the subblock size should be selected by balancing approximation accuracy and computational efficiency. The spatial-frequency samples of the (*i*, *j*)th subregion can be expressed as follows:

(17) $${S^{\prime }}_{i,j}\left(f\right)=\sum \sigma \left(x\prime ,y\prime \right)e^{-j2\pi \left(k_{x}x\prime +k_{y}y\prime \right)}$$

where *x*′ and *y*′ denote the coordinates of the target scatterers within the local subblock. The spatial-frequency samples of the (*i*, *j*)th subblock are filled into an independent spatial-spectrum matrix *H_i,j_* according to their corresponding spatial-frequency coordinates. An inverse Fourier transform is then performed to obtain the corresponding sub-image:

*Image_i_*_,*j*_(*x*′,*y*′) = *IFFT*(*H_i_*_,*j*_) (18)

For each sub-image *Image_i_*_,*j*_(*x*′, *y*′), whose local coordinate system has its origin at (*x_i,j_^origin^*, *y_i,j_^origin^*) in the global image plane, the local coordinates must be transformed and mapped back to the global coordinate system. The coordinate transformation from the (*i*, *j*)th sub-image to the global image can be expressed as

(19) $$\{\begin{array}{l} x=x_{i,j}^{\mathrm{origin}}+x^{\prime }\\ y=y_{i,j}^{\mathrm{origin}}+y^{\prime } \end{array}$$

After all sub-images are stitched together, the complete image of the full imaging region can be represented as

(20) $$\mathrm{Image}\left(x,y\right)=\{\begin{array}{ll} {\mathrm{Image}}_{i,j}\left(x-x_{i,j}^{\mathrm{origin}},y-y_{i,j}^{\mathrm{origin}}\right), & \mathrm{if}\;\left(x,y\right)\in \mathrm{region}\left(i,j\right)\\ 0, & \mathrm{otherwise} \end{array}$$

where *i* ∈ {1, …, *M*} and *j* ∈ {1, …, *N*}. The subblock indices identify the location of each local image in the global coordinate system. After coordinate scaling and stitching, the final image contains the scatterers across the full region.

### 2.2. Block-Segmented Imaging Procedure

**Figure 2:**
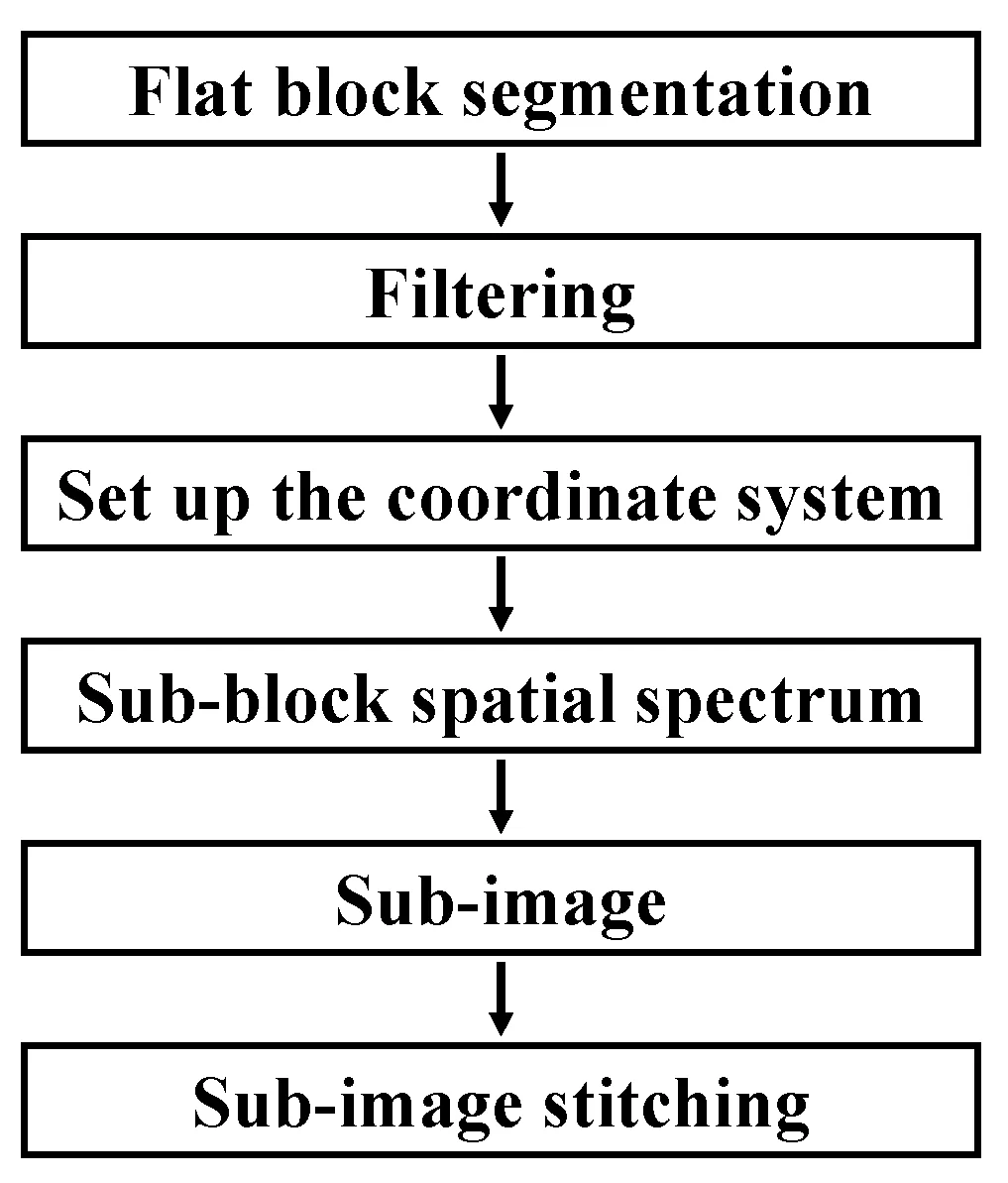
Processing flow of the block-segmented imaging method.

Figure 2 summarizes the processing workflow of the proposed block-segmentation imaging method, in which spatial-spectrum construction is the core step for image reconstruction. Specifically, the entire imaging plane (target area) is first divided into several subblocks, and the received target echoes are subjected to digital bandpass filtering to suppress out-of-band interference signals. Each subblock establishes its own local coordinate system and performs a Fourier transform on the echoes collected by the transmit–receive antenna array within the subblock’s local coordinate system to obtain complex frequency-domain echo data. Subsequently, using the local coordinate origin of each subblock as a reference, propagation phase compensation is applied to the complex frequency-domain echo data to obtain complex measurement values, thereby establishing a correspondence between the complex measurement values and the spatial spectrum of the subblocks under conditions of local linear phase approximation. Based on the positions of the transmit and receive antennas and the echo frequencies, the two-dimensional spatial-frequency coordinates corresponding to each complex measurement value are determined and mapped to the corresponding locations in the spatial-frequency grid to construct the spatial-spectrum matrices for each subblock. Finally, the individual sub-images are generated using a two-dimensional inverse Fourier transform, mapped back to the global coordinate system, and stitched together to form the complete target image. Because every scatterer is close to at least one local origin, the locally linear phase approximation remains valid over a larger global field of view. The procedure is also compatible with parallel implementation on GPUs or FPGAs because each subblock can be reconstructed independently before stitching.

The proposed block-segmented imaging procedure is a computational reconstruction strategy, rather than a measurement procedure performed based on different physical coordinate systems. The subblocks are partitions of the imaging plane or target scene. Only a single complete acquisition of the multi-antenna echo data is required. The same data are reused for reconstructing all subblocks, eliminating the need for separate measurements for individual subblocks. The full imaging plane is divided in post-processing into several local subblocks. This also constitutes one of the main contributions: introducing local coordinate models for computational subblocks to reduce higher-order propagation phase errors and extend the validity of linear spatial-frequency mapping, thereby enabling wide-area near-field spatial-spectrum imaging.

## 3. Simulation Verification

### 3.1. Imaging-Field Extension with Point Targets

**Figure 3:**
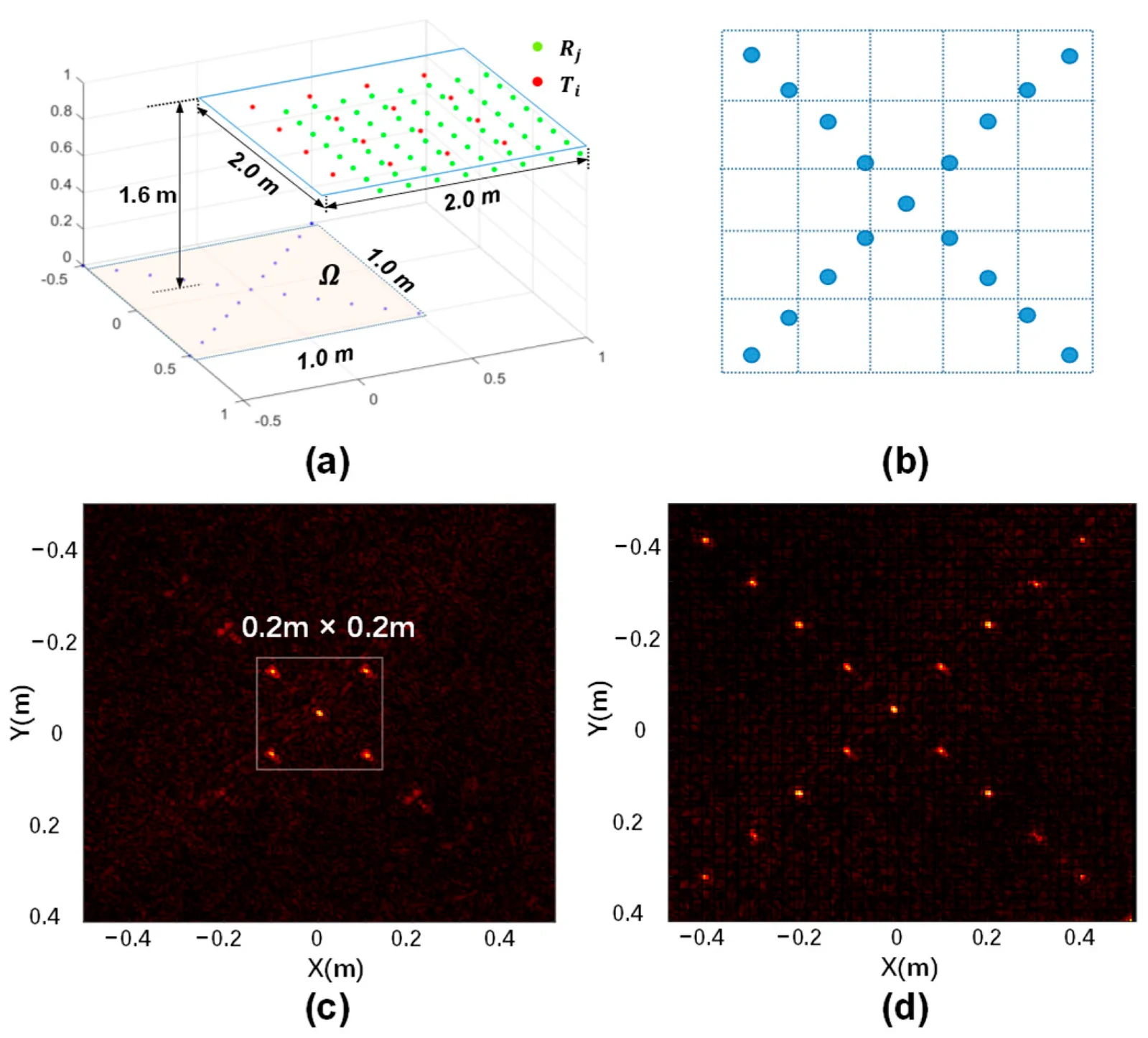
Verification of block-segmented imaging with point targets. (**a**) Simulation geometry with a 2 m × 2 m transmit/receive aperture and a 1 m × 1 m imaging plane, green and red markers denote receive and transmit antenna positions, respectively. (**b**) Point-target distribution and 25-subblock partition. (**c**) Direct reconstruction without block segmentation, where only a local 0.2 m × 0.2 m region is well-focused. (**d**) Reconstruction after block segmentation and smooth stitching over the full 1 m × 1 m imaging plane.

The first simulation evaluates the capability of block segmentation to extend the effective imaging area under a fixed antenna aperture. As shown in Figure 3a, the transmit and receive antennas are distributed over a 2 m × 2 m aperture located 1.6 m from the 1 m × 1 m imaging plane. A cross-shaped point-target pattern is arranged on the imaging plane, and the subblock layout used for local reconstruction is shown in Figure 3b. A 32–38 GHz linear frequency-modulated (LFM) signal with a pulse width of 4 μs is used. Without block segmentation, the far-field linear approximation is valid only within a local region of approximately 0.2 m × 0.2 m around the global coordinate origin. As shown in Figure 3c, scatterers outside this local region are defocused or weakly reconstructed because the accumulated high-order phase error invalidates the single-origin spatial-frequency mapping. The 1 m × 1 m imaging plane is then divided into 25 subblocks, with the center of each subblock defined as an independent local coordinate origin. Local spatial-spectrum imaging is performed for each subblock, followed by sub-image stitching. As shown in Figure 3d, the point targets over the entire 1 m × 1 m region are effectively reconstructed. These results verify the basic mechanism of the proposed method: block segmentation reduces the spatial offsets of scatterers relative to their local origins, makes high-order nonlinear phase terms negligible within each subblock, restores local linear spatial-frequency mapping, and enables large-area near-field imaging through sub-image stitching.

### 3.2. Complex Extended-Target Simulation

**Figure 4:**
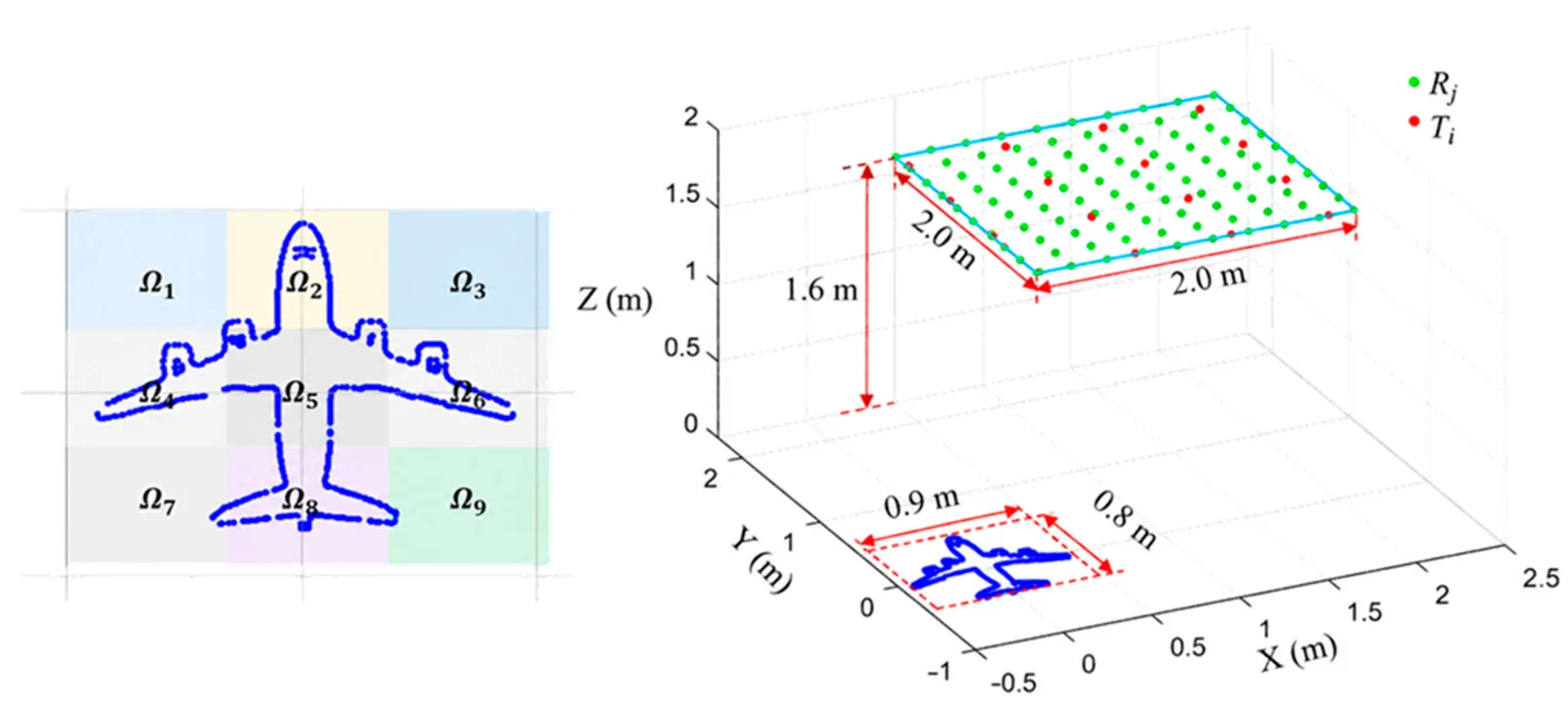
Complex extended-target simulation scenario. The aircraft-shaped target is partitioned into nine subregions Ω1–Ω9, and the 2 m × 2 m transmit/receive aperture is located 1.6 m above the target plane.

A second simulation employs an aircraft-shaped complex target to further verify the proposed method. As shown in Figure 4, the aircraft-shaped target is divided into nine local regions, denoted as Ω1–Ω9, and the transmit and receive antennas are distributed over a 2 m × 2 m square aperture located 1.6 m above the target plane. The imaging region is partitioned into non-overlapping subblocks at uniform spatial intervals, with the coordinate ranges and boundaries of each subblock predefined. The simulation uses a 27–31 GHz Ka-band signal, and the target occupies an area of approximately 0.9 m × 0.8 m. Under this imaging geometry, different target regions exhibit large spatial offsets relative to a single global coordinate origin, causing the far-field approximation to gradually become invalid and resulting in defocus in the global spatial-spectrum image. Therefore, an independent local coordinate origin is assigned to each subblock, and spatial-spectrum matrix construction and image inversion are performed separately within the corresponding local coordinate system.

**Figure 5:**
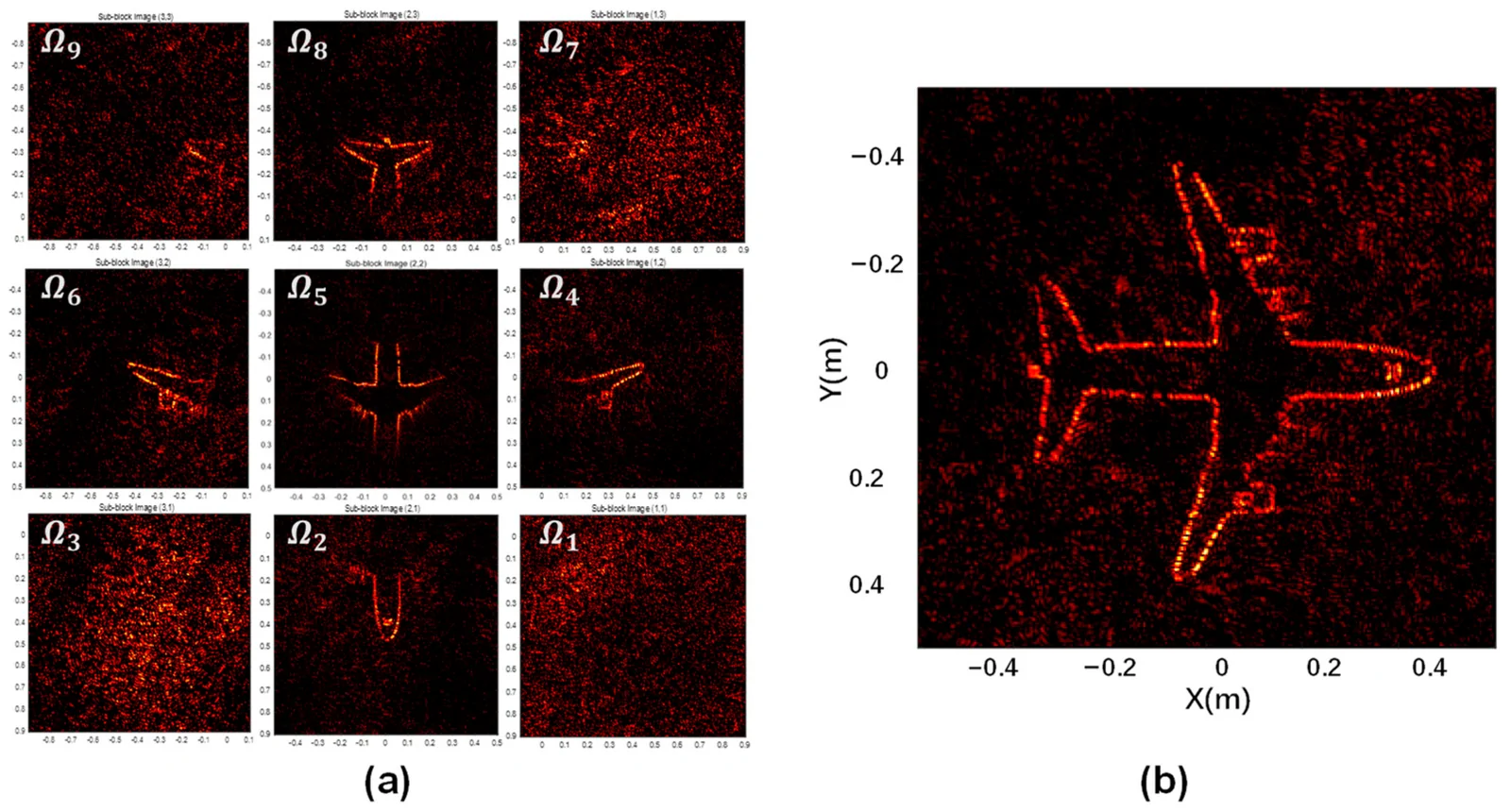
Imaging of an aircraft-shaped extended target. (**a**) Sub-images corresponding to the nine local regions. (**b**) Stitched full image after coordinate mapping and smooth stitching.

Figure 5a presents the imaging results of the nine individual subblocks, with the center of each subblock defined as the origin of its local coordinate system. Each subblock is reconstructed with respect to its local origin. Scatter points within the subblock satisfy the local linear phase approximation and can be effectively focused, whereas scatter points outside the subblock and farther from its local origin may be poorly focused because of higher-order phase errors. During stitching, only pixels within each subblock’s predefined coordinate range are retained and mapped to their corresponding positions in the global image. This coordinate-range restriction also applies when smaller subblocks are used to improve the accuracy of the local approximation. Consequently, each global pixel is assigned to exactly one subblock, preventing duplicated regions or gaps caused by repeated placement of sub-images or omission of boundary pixels. For more complex scenarios in the future, boundary continuity can be further improved by combining weighted fusion with consistency checks for overlapping regions [20]. The nine sub-images reconstruct different local regions of the aircraft-shaped target and therefore provide complementary contour information. When a single global coordinate system is used, the global origin is located within the region corresponding to Ω5, and the resulting image is similar to the local reconstruction of Ω5, revealing only part of the aircraft contour. After each sub-image is mapped to the global coordinate system and smoothly stitched, the complete aircraft contour is obtained, as shown in Figure 5b. To further quantify image quality, the mean squared error (MSE) is introduced to measure the discrepancy between the reconstructed image and the true target scattering model. It is defined as

(21) $$\mathrm{MSE}=\frac{1}{M\times N}\sum_{i=0}^{M-1}\sum_{j=0}^{N-1}{\left[I_{\mathrm{true}}\left(i,j\right)-I_{\mathrm{image}}\left(i,j\right)\right]}^{2}$$

where *I_true_*(*i*,*j*) denotes the scattering intensity of the true aircraft scattering model at pixel location (*i*, *j*), *I_image_*(*i*,*j*) denotes the corresponding pixel value in the reconstructed image, and *M* × *N* represents the image dimensions in pixels. Based on this metric, the MSEs of the single-global-coordinate imaging result and the block-segmented imaging result are calculated with respect to the true scattering model. The resulting MSEs are 0.34 and 0.08, respectively. The MSE of the block-segmented image is reduced by approximately 76.5% compared with that of the single-global-coordinate image, demonstrating that the proposed method significantly reduces imaging distortion caused by near-field nonlinear phase errors and improves the overall image quality of extended targets.

## 4. Experimental Results

### 4.1. Ka-Band Microwave-Photonic Imaging Prototype

To verify the effectiveness of the proposed method, a Ka-band near-field imaging testbed is built using discrete microwave and photonic components. The system mainly consists of an RF signal generation/transmission module and a microwave-photonic broadband reception module. To suppress multipath reflections from walls and the tabletop in the experimental environment, absorbing materials are placed around the target area. The experiment emulates a 3-transmit/13-receive multi-antenna array, forming 39 transmit–receive pairs. In our laboratory prototype, a 3-transmit/13-receive multi-antenna aperture is emulated by time-division movement because only one Ka-band RF transmit chain and one receive chain are available. After echo acquisition at each transmit–receive position pair, the antennas are sequentially repositioned to the next prescribed pair until data from all transmit–receive position combinations are collected. This time-division implementation is only an experimental acquisition scheme. In future implementations, a fixed array comprising three transmit antennas and 13 receive antennas can enable single-snapshot data acquisition, provided that sufficient RF transmit and receive chains are available.

**Figure 6:**
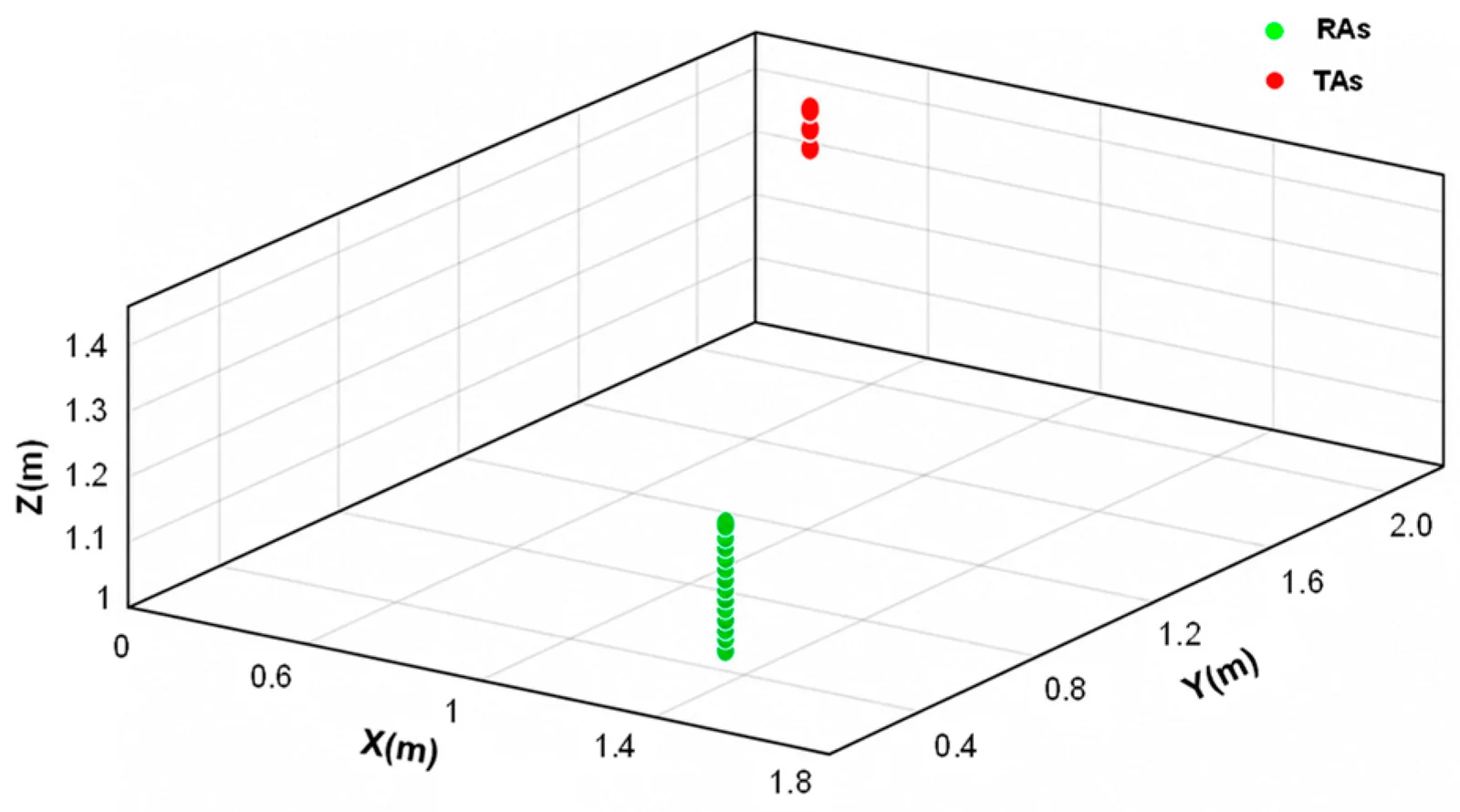
Experimental antenna arrangement for the emulated 3-transmit/13-receive multistatic aperture. Red markers denote the transmit antenna positions, green markers denote the receive antenna positions, and the reconstructed target scene is located on the YOZ imaging plane.

Regarding the antenna arrangement, the design objective is to maximize two-dimensional spatial-spectrum coverage of the target area while ensuring sufficient SNR and maintaining controllable hardware complexity. The optimal antenna arrangement depends on the target size, detection range, desired spatial-frequency coverage, antenna beamwidth, and hardware cost. This study employs a 3-transmit/13-receive configuration as a compact experimental aperture for proof-of-concept testing in close-range security screening; this does not represent a globally optimal arrangement. To reduce positioning errors caused by inaccurate lateral movement, the antenna positions are mainly adjusted in the vertical direction by changing the tripod height, thereby forming an equivalent two-dimensional planar aperture that is approximately perpendicular to the *XOY* plane. The *YOZ* plane is used as the imaging plane. The experimental antenna arrangement is shown in Figure 6. The transmitting antennas are approximately 0.5 m from the imaging plane, with a vertical spacing of approximately 0.03 m between adjacent transmitting antennas. The receiving antennas are approximately 1.3 m from the imaging plane, with a vertical spacing of approximately 0.015 m between adjacent receiving antennas.

**Figure 7:**
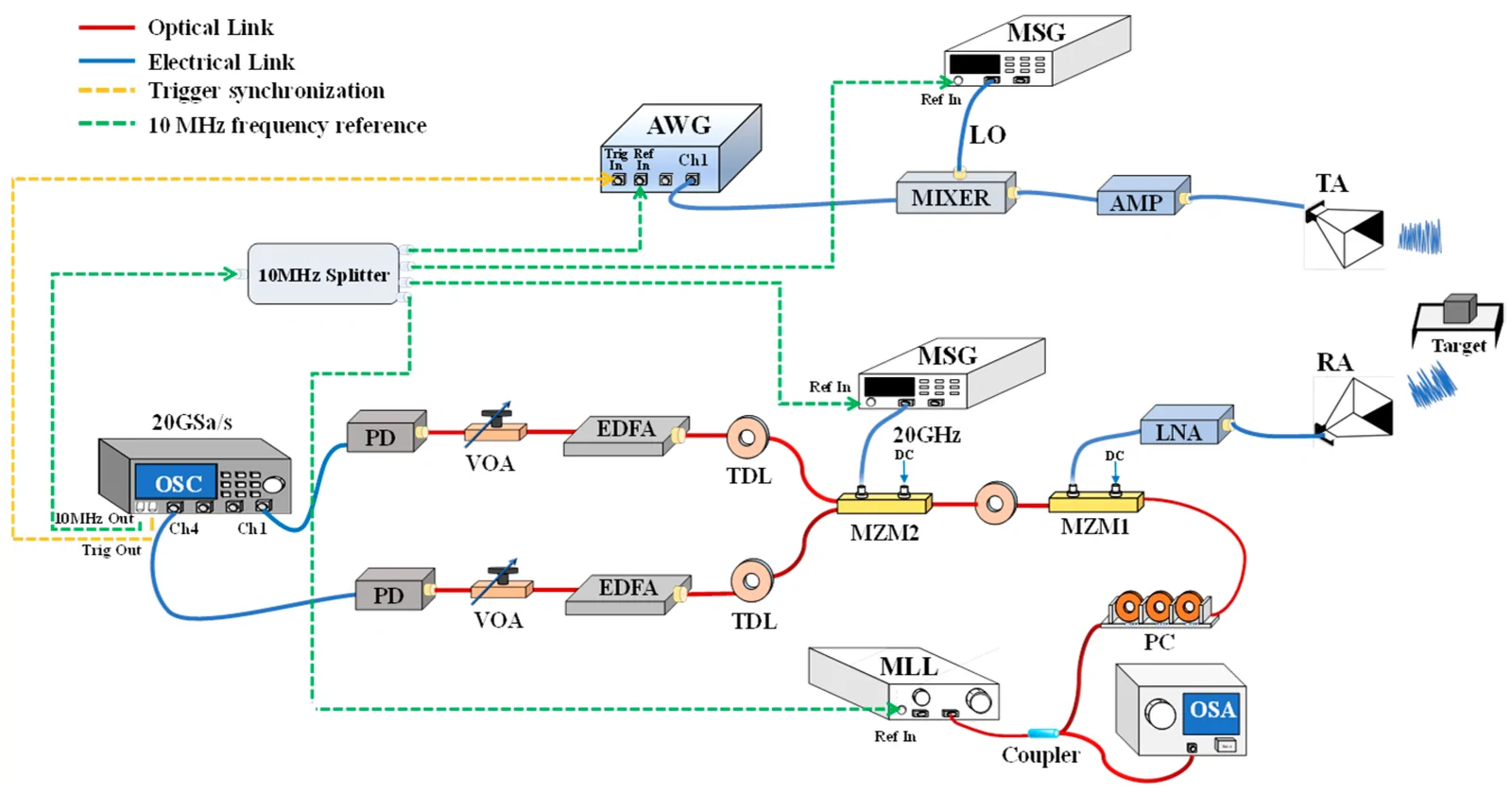
Experimental setup for block-segmented near-field imaging with microwave-photonic broadband reception. The system includes RF signal generation and upconversion, Ka-band transmission and reception, electro-optic sampling, optical demultiplexing, PD, and two-channel OSC acquisition.

The system-level signal chain is shown in Figure 7. An LFM signal with a frequency range of 2–8 GHz is generated in MATLAB R2024a (MathWorks, Natick, MA, USA) and loaded into an arbitrary waveform generator (AWG, Keysight M8195A, Keysight Technologies, Santa Rosa, CA, USA). The intermediate-frequency signal generated by the AWG is delivered through a coaxial cable to a mixer (Marki MM1-1140H, Marki Microwave, Morgan Hill, CA, USA), where it is mixed with a 30 GHz continuous-wave local oscillator generated by a microwave signal generator (MSG, Keysight N5166B, Keysight Technologies, Santa Rosa, CA, USA), thereby upconverting the LFM signal to the 32–38 GHz Ka band. The upconverted signal is amplified by a power amplifier (AMP, CONNPHY CMP-18G-40G-3021-K, Connphy Microwave Inc., USA) and fed to a transmit horn antenna (TX, INFOMW LB-28-25-C2-KF, AINFO, Irvine, CA, USA) for radiation toward the target region. The target echoes are received by a horn antenna (RX, INFOMW LB-28-25-C2-KF), amplified by a low-noise amplifier (LNA, TLLA26G40G-35-50, Talent Microwave, Suzhou, China), and then delivered to the microwave-photonic broadband reception module. In this module, a mode-locked laser (MLL, PSL-40-TT, Calmar Laser, Palo Alto, CA, USA) generates a 40 GHz optical pulse train, which is split into two branches by a 3-dB dual-output optical coupler. One branch is connected to an optical spectrum analyzer to monitor the mode-locking state and optical pulse quality, while the other passes through a polarization controller to optimize the polarization state for subsequent electro-optic modulation. The optical pulse train is fed into a Mach–Zehnder modulator for electro-optic sampling (MZM1, EOSPACE AX-0MVS-40-PFA-PFA-LV, EOSPACE, Redmond, WA, USA), where the LNA-amplified Ka-band echo is applied to the RF port. The modulated optical signal then passes through a tunable delay line and enters a second-stage Mach–Zehnder modulator (MZM2, EOSPACE AX-1 × 2-0MxSS-20-SFU-LV, EOSPACE, Redmond, WA, USA), which operates as a parallel demultiplexing device driven by a 20 GHz single-tone signal output from the MSG. The two complementary outputs of MZM2 are connected to tunable optical delay lines (TDLs), where the relative delay between the two channels is adjusted to 25 ps to ensure correct temporal interleaving and suppress reconstruction spurs caused by channel timing mismatch. The two optical signals are amplified by erbium-doped fiber amplifiers (EDFA) and adjusted by variable optical attenuators to approximately 0 dBm before photodetection to avoid detector saturation. Finally, the two electrical outputs from the photodetectors (PD, CONQUER KG-PT-10G-SM-FA, CONQUER, Beijing, China) are digitized by the CH1 and CH4 channels of an oscilloscope (OSC, Keysight DSO-S 804A, Keysight Technologies, Santa Rosa, CA, USA), and the two-channel data are reordered and interleaved in MATLAB to reconstruct the broadband RF echo signal. The experiment utilizes standard Ka-band horn antennas, with the beam directed toward the target area to be imaged. Although the antenna gain is relatively low in regions away from the beam center, the echo SNR is improved through power amplification at the transmitter and low-noise amplification at the receiver, thereby generating echoes that are still usable for imaging. The 39 transceiver observation geometries, formed by three transmitting positions and 13 receiving positions, achieve full coverage of the target area.

During data acquisition, the various instruments must be synchronized using a unified trigger signal and frequency reference. Specifically, the OSC Trigger out port is connected to the AWG Trigger in port via a coaxial cable, while the OSC 10 MHz out port is connected to the AWG Ref_clk port via another coaxial cable. The AWG is configured to use an external reference clock and operate in single-trigger output mode. In addition, the OSC 10 MHz reference output is distributed through a multiport splitter and connected via coaxial cables to the external reference inputs of the two MSGs and the MLL, allowing these instruments to share a common frequency reference. Due to experimental limitations, we utilize a single transmit and receive chain, sequentially acquiring 39 echo datasets (from three transmit and 13 receive positions) via time-division acquisition. The imaging results derived from these data demonstrate that the phase consistency across individual measurements satisfies the requirements for coherent imaging under the experimental conditions.

The Ka-band imaging system is used to verify the capability of the proposed block-segmented spatial-spectrum method for large-area, high-resolution near-field imaging under broadband operation. The operating frequency is set to 32–38 GHz. On the one hand, the 6 GHz instantaneous bandwidth expands the two-dimensional spatial-frequency support and increases the spatial bandwidth along two orthogonal directions, thereby improving the two-dimensional imaging resolution. On the other hand, this frequency band is matched to the 40 GHz optical pulse sampling rate of the microwave-photonic broadband acquisition module. Through photonic bandpass sampling and equivalent frequency downconversion, the 32–38 GHz Ka-band echoes are mapped to the 2–8 GHz band, which falls within the 8.4 GHz analog bandwidth of the electronic acquisition chain, enabling direct digitization of broadband Ka-band echoes.

### 4.2. Broadband Spatial-Spectrum Imaging Resolution Validation

**Figure 8:**
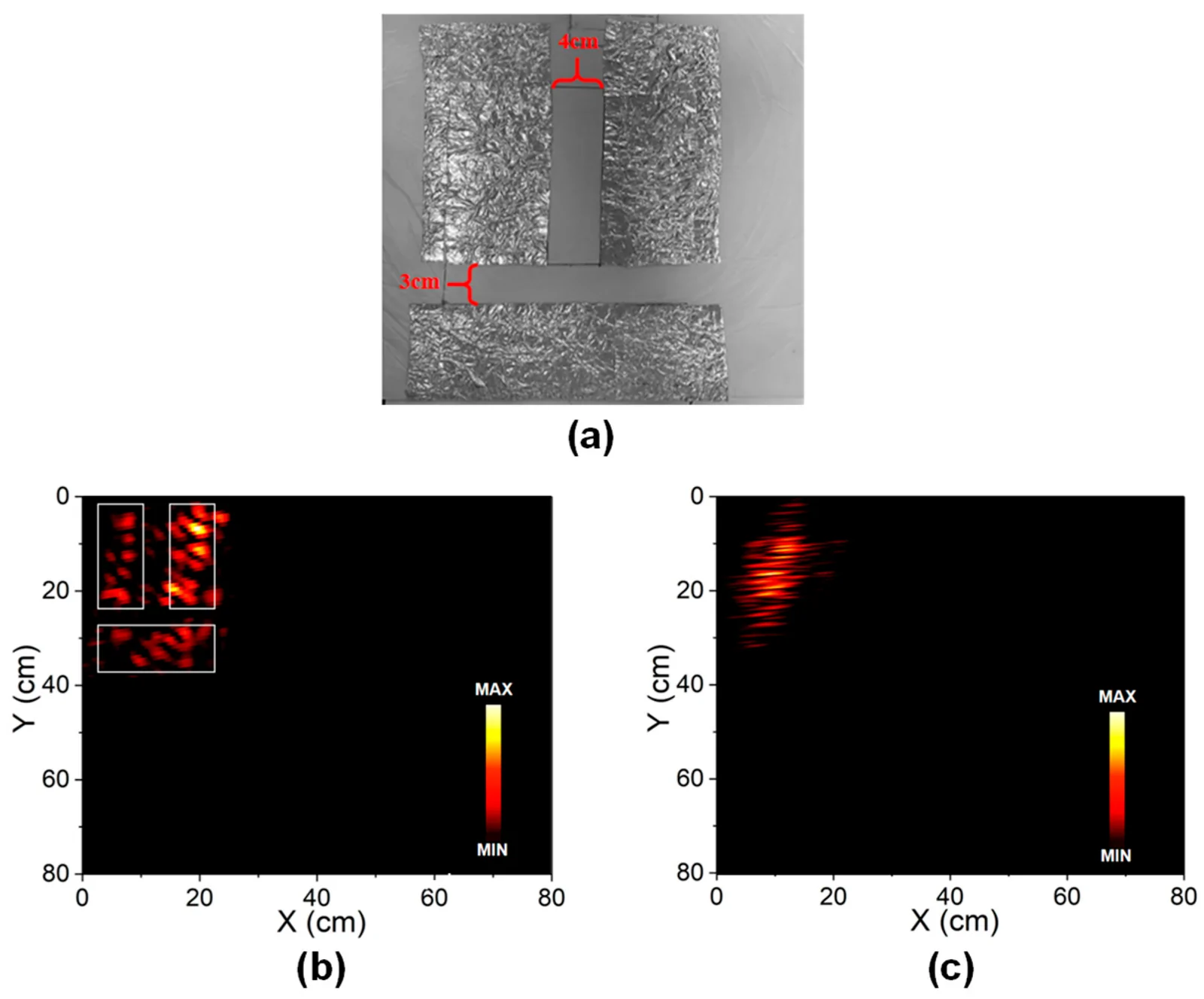
Resolution test under different bandwidths. (**a**) Aluminum-foil resolution target with 4 cm horizontal spacing and 3 cm vertical spacing. (**b**) Image reconstructed with the full 6 GHz bandwidth, showing clear separation of the aluminum-foil strips. (**c**) Image reconstructed with a reduced 2 GHz bandwidth, showing degraded edge separation and stronger blur.

The imaging resolution under the current array configuration and the improvement provided by increased signal bandwidth are experimentally evaluated. Based on the antenna array geometry and the 32–38 GHz operating frequency band, the spatial bandwidth, defined as the difference between the maximum and minimum spatial frequencies in each direction, is calculated in MATLAB along two orthogonal directions. The theoretical two-dimensional imaging resolution, given by the reciprocal of the spatial bandwidth, is approximately 3 cm × 4 cm. In this experiment, resolution test targets are constructed using aluminum-foil strips arranged along two orthogonal directions, inspired by the use of stripe patterns with known dimensions in the U.S. Air Force’s 1951 resolution target [21]. To verify the theoretical result, three aluminum-foil strips, each measuring 20 cm × 8 cm, are attached to a foam board to form a resolution test target, as shown in Figure 8a. Along the *x*-direction, the two vertically oriented strips are separated by 4 cm. Along the *y*-direction, the horizontal strip is separated from the two vertical strips by 3 cm. The two-dimensional resolution capability is evaluated by determining whether the scattering regions corresponding to adjacent strips can be distinguished from one another. The coordinate origin of the overall imaging region is placed near the upper-left corner of the first aluminum strip so that the dominant scattering region remains within the valid range of the linear spatial-frequency approximation. Since the target spacing is designed according to the theoretical resolution under a 6 GHz bandwidth, the full 32–38 GHz band is first used to verify the system resolution. As shown in the white-boxed regions of Figure 8b, the contours of the three aluminum strips and multiple scattering points within them are clearly reconstructed, and distinct edge separation is observed between adjacent strips. This demonstrates that the 6 GHz broadband signal provides centimeter-level two-dimensional resolution consistent with the theoretical analysis. By measuring the spacing between adjacent resolvable scattering regions along the *x*- and *y*-directions in Figure 8b, the experimental imaging resolution is approximately 3.3 cm × 4.7 cm, which is on the same centimeter scale as the theoretical value of 3 cm × 4 cm [22]. The discrepancy mainly arises from nonideal system effects, including antenna-position errors and random scattering from the crumpled aluminum-foil surfaces. Although antenna positioning errors were minimized as much as possible through calibration, unavoidable residual deviations still exist, resulting in the actual spatial-frequency coverage not fully aligning with the theoretical calculations. To further verify the effect of signal bandwidth on spatial-spectrum imaging resolution, only the echo data corresponding to a 2 GHz instantaneous bandwidth are extracted from the original measurements for spatial-spectrum imaging, while the array aperture and measured dataset remain unchanged. Therefore, no additional LFM waveform generation or data acquisition is required. As shown in Figure 8c, when the bandwidth is reduced, adjacent aluminum strips and their scattering points exhibit obvious overlap, making the target edges difficult to distinguish. This occurs because reducing the signal bandwidth compresses the two-dimensional spatial-frequency support and decreases the available spatial bandwidth. According to spatial-spectrum imaging theory, imaging resolution is inversely proportional to spatial bandwidth; therefore, reduced spatial-frequency support directly degrades the two-dimensional imaging resolution.

In the present two-dimensional spatial-spectrum formulation, the role of bandwidth is broader because the spatial-frequency coordinates are proportional to both RF frequency and antenna look direction. The increase in bandwidth broadens the spatial-frequency support, thereby increasing the available spatial bandwidth along the two orthogonal imaging directions. The reported 3.3 cm × 4.7 cm value is therefore the measured imaging resolution obtained from the separability of adjacent scattering regions in Figure 8b, rather than merely the physical size of the aluminum-foil target.

The radar images in Figure 8 should be interpreted as scattering-intensity distributions rather than optical photographs. The full-bandwidth reconstruction resolves the designed 3 cm and 4 cm feature separations through distinguishable scattering regions and edge separation, whereas the reduced-bandwidth reconstruction merges adjacent scattering features because the spatial-frequency support is compressed.

These experimental results indicate that, in small-scale array scenarios such as security screening, the achievable resolution is constrained by the limited physical aperture, and simply enlarging the array aperture is of limited practicality. By introducing broadband spectrum resources, the two-dimensional spatial-frequency support can be further expanded, thereby compensating for the insufficient resolving capability caused by the limited physical aperture and improving the two-dimensional resolution of near-field imaging. Meanwhile, microwave-photonic broadband reception provides an effective hardware solution for overcoming the bandwidth limitations of electronic receiver chains, enabling direct acquisition of broadband millimeter-wave echoes and supporting high-resolution spatial-spectrum imaging [13,14,15].

### 4.3. Extended-Area Imaging of a Gun-Shaped Threat Analog

**Figure 9:**
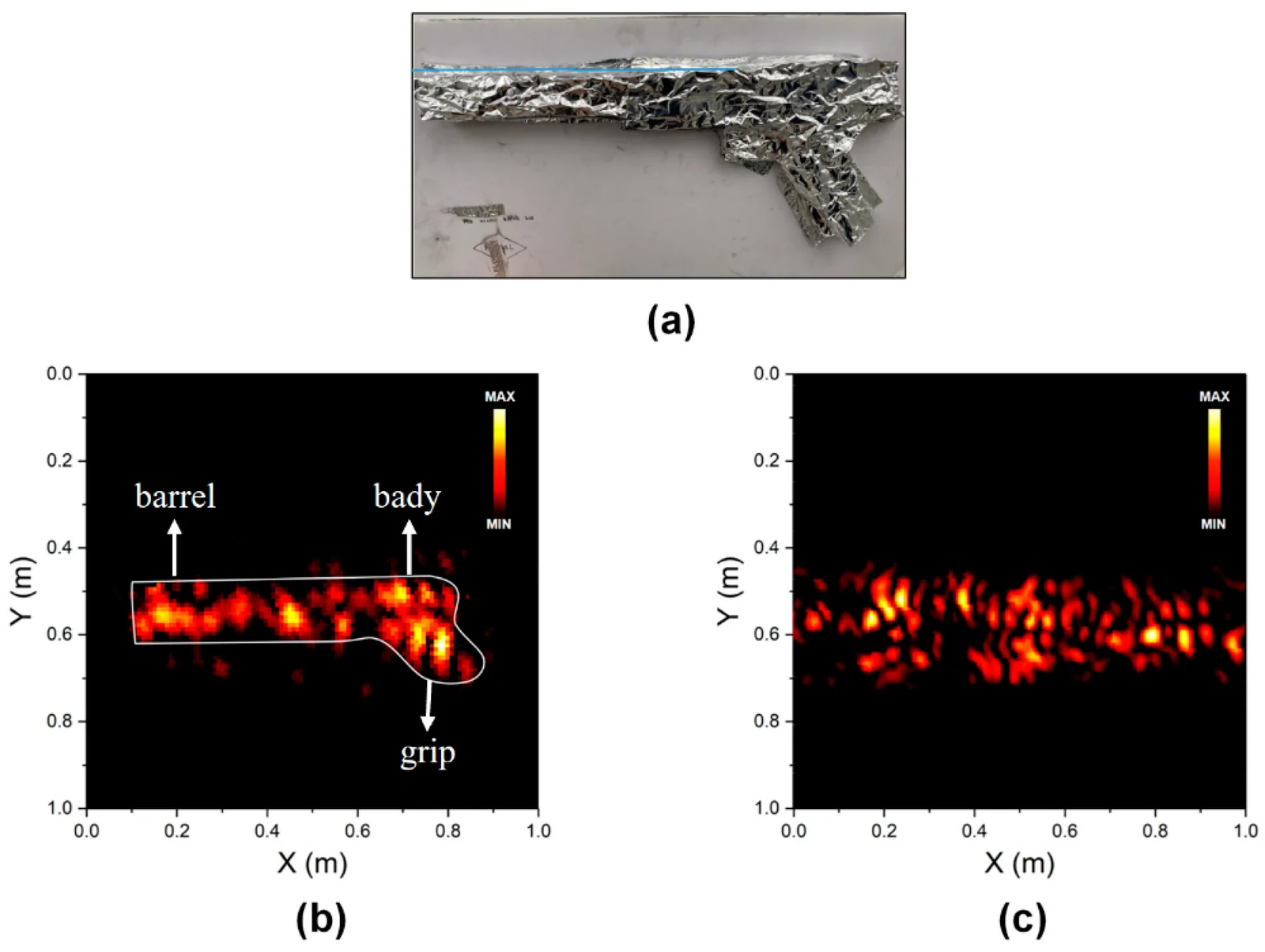
Experimental gun-shaped target. (**a**) Photograph of the aluminum-foil gun-shaped target. (**b**) Reconstruction with block segmentation, where the main body, barrel, and grip are recognizable. (**c**) Reconstruction without block segmentation, showing discontinuous bright spots and severe defocus.

To verify the capability of the proposed block-segmentation method to extend the effective near-field imaging area, a gun-shaped target with an approximate size of 80 cm × 25 cm is fabricated using aluminum foil, as shown in Figure 9a. This target is used for proof-of-principle validation of imaging complex extended targets, rather than the identification of actual firearms. Owing to the relatively large target size and the short-range, small-scale array configuration, even when the target is located near the center of the imaging region, scatterers far from the global coordinate origin may no longer satisfy the far-field linear approximation, leading to non-negligible high-order nonlinear phase errors and image defocusing. To further enhance this effect and provide a more direct validation of the proposed method, the global coordinate origin is shifted away from the target and placed approximately 10 cm along the *x*-direction and 40 cm along the *y*-direction from the leftmost point of the target, such that the target as a whole is located farther from the global origin. Under this initial global coordinate system, scatterers at different locations on the target cannot simultaneously satisfy the linear spatial-frequency approximation associated with a single coordinate origin.

To address the above issue, the 1 m × 1 m imaging plane is divided into 25 subblocks, each measuring 20 cm × 20 cm. An independent local coordinate system is established for each subblock, with the center of each subblock serving as the local coordinate origin. First, the phase-compensation factor is constructed according to the propagation distances from the transmit and receive antennas to the coordinate origin of each subblock. Subsequently, the mapping relationship between spatial frequency and target spatial coordinates is established. Based on this mapping, the spatial-spectrum matrix of each subblock is extracted, and the corresponding sub-image is obtained through an inverse Fourier transform. Finally, all sub-images are mapped from their local coordinate systems back to the global imaging plane and stitched together, yielding the complete 1 m × 1 m reconstructed image. As shown in the white-boxed region of Figure 9b, after block segmentation, the overall contour of the gun-shaped target, including the main body, barrel, and grip, can be clearly identified, and the reconstructed dimensions are consistent with the preset target geometry. In contrast, the reconstruction without block segmentation is shown in Figure 9c. Because the target is relatively large and located far from the global coordinate origin, some scatterers are significantly affected by high-order nonlinear phase terms, resulting in spatial-frequency mapping mismatch in the spatial-spectrum matrix and ultimately causing contour loss and severe defocusing. These experimental results demonstrate that block segmentation reduces the spatial offsets of scatterers relative to their local origins by establishing independent local coordinate origins for different target regions. This suppresses high-order nonlinear phase errors, restores local linear spatial-frequency mapping, and ultimately enables imaging inversion of large-area near-field targets through sub-image stitching.

For the 32–38 GHz experimental band, the wavelength ranges from approximately 9.38 mm to 7.89 mm. Thus, the 1 m × 1 m imaging plane corresponds to approximately 107λ–127λ in linear size, the 80 cm × 25 cm gun-shaped target corresponds to approximately 85λ × 27λ at 32 GHz and 101λ × 32λ at 38 GHz, and each 20 cm × 20 cm subblock corresponds to approximately 21λ–25λ. The proposed method is applicable to large near-field targets whose overall extent does not satisfy the far-field approximation with respect to a single global origin. Scattering points far from this origin introduce substantial higher-order propagation phase errors, making a single global linear phase approximation inadequate for the entire target. As long as the local linear phase approximation holds within each subblock, the method can improve the focusing performance for different target regions. Small targets located entirely near the global origin can generally be imaged effectively without segmentation. Therefore, the method does not provide the same improvement for targets of all sizes.

The reconstructed radar image of the gun-shaped target also represents its scattering-intensity distribution rather than a continuous optical contour. Because metallic surfaces exhibit directional specular reflection, local surfaces whose reflections are directed toward the receiving aperture typically produce strong bright spots, whereas those whose reflections are directed away may yield weak echoes, leaving portions of the contour indistinct or missing. To mitigate this effect, the aluminum-foil surface is deliberately wrinkled to create multiple local scattering facets with different orientations, increasing the number of observable scattering points. Similar specular-reflection issues arise in optical imaging of metallic objects [23]. However, optical highlight removal primarily addresses details obscured by strong reflections, whereas the missing contour segments discussed here may result from weak echoes associated with unfavorable reflection directions. The proposed block-segmented method reduces higher-order near-field propagation phase errors and improves the focusing of the acquired scattering information, but it cannot recover scattering information that is not effectively received. Therefore, the completeness of the reconstructed target contour remains jointly affected by angular coverage, polarization, material scattering properties, and antenna beam illumination.

## 5. Discussion

Compared with the conventional time-domain BP algorithm for near-field imaging, the proposed method offers advantages mainly in terms of computational efficiency and the utilization of broadband spectral resources. The computational efficiency of the proposed method does not depend directly on the absolute size of the target, but rather on the number of subblocks required to cover the target and the FFT size used for each local reconstruction. BP is based on the exact spherical-wave propagation distance and requires point-by-point phase compensation and coherent accumulation over every imaging pixel, transmit–receive channel, and frequency sample. If the entire imaging region is divided into *M* × *N* equivalent regions, with each region containing *P_x_* × *P_z_* reconstruction pixels, the computational complexity of BP is approximately *O*(*MNP_x_P_z_N_c_N_f_*), where *N_c_* and *N_f_* denote the numbers of transmit–receive channels and frequency samples, respectively. In contrast, the proposed method maps the broadband echoes onto the two-dimensional spatial-frequency domain and reconstructs each subblock through a two-dimensional fast Fourier transform, resulting in a dominant computational complexity of approximately *O*[*MNP_x_P_z_*log_2_(*P_x_P_z_*)]. This avoids the pixel-by-pixel multiplication between the image dimension and the channel–frequency sampling dimension required by BP, thereby substantially reducing the computational burden for broadband multichannel imaging. In addition, in conventional range–cross BP imaging, the resolution improvement provided by increased signal bandwidth is typically manifested primarily in the range dimension, whereas the cross-range resolution is mainly constrained by the physical aperture and center wavelength. In the proposed method, echoes corresponding to different transmit positions, receive positions, and frequencies are directly mapped onto the two-dimensional spatial-frequency distribution of the target imaging plane. Consequently, the broadband spectral resources provided by microwave-photonic reception expand the spatial-frequency coverage along both orthogonal imaging directions, thereby improving the two-dimensional imaging resolution under limited-aperture conditions. In addition, compared with conventional spatial-spectrum imaging methods, the proposed block-segmentation strategy extends the effective imaging region from a local area of approximately 0.2 m × 0.2 m to the full 1 m × 1 m imaging plane, corresponding to a fivefold increase in linear imaging span and a 25-fold increase in imaging area. This effectively alleviates the defocusing of scatterers far from the global coordinate origin caused by the breakdown of the far-field approximation. Overall, through the joint use of block-segmented spatial-spectrum processing and microwave-photonic broadband reception, the proposed method simultaneously achieves centimeter-level imaging resolution, extended near-field imaging coverage, and high computational efficiency with a relatively small antenna array, providing a feasible technical solution for millimeter-wave security-screening imaging.

It should also be emphasized that the antenna beamwidth and angular coverage affect the actual spatial-frequency support that can be obtained [24]. The spatial frequency is jointly determined by the transceiver observation geometry and the signal frequency, whereas the antenna beamwidth dictates whether the corresponding measurement samples can be effectively utilized by affecting the echo amplitude and SNR. As long as the target area is covered by the transceiver beams and the echo SNR satisfies the imaging requirements, the available spatial-frequency coverage is mainly dictated by the observation angular coverage and the signal frequency band. The experiment utilizes standard Ka-band horn antennas, with the beam directed toward the target area to be imaged. Although the antenna gain is relatively low in regions away from the beam center, the echo SNR is improved through power amplification at the transmitter and low-noise amplification at the receiver, thereby generating echoes that are still usable for imaging. The 39 transceiver observation geometries, formed by three transmitting positions and 13 receiving positions, achieve full coverage of the target area. The synthesized beam formed by the transceiver combination’s effective aperture determines the angular resolution of conventional beamforming imaging at a given frequency. In this paper, we employ spatial-spectrum computational imaging, which jointly maps the echoes from different transceiver positions and frequencies into the spatial spectrum. The signal bandwidth extends the spatial frequency coverage in both imaging dimensions. Therefore, at the same beamwidth, bandwidth expansion can achieve higher imaging resolution.

Table 1 compares the main performance metrics of the proposed method with representative existing imaging approaches [16,17]. Because these methods differ in array scale, imaging principle, operating scenario, and experimental conditions, the comparison is intended primarily to illustrate their characteristics in terms of resolution, system scale, and computational efficiency. With a 3 × 13 transmit–receive configuration, the proposed prototype achieves a measured resolution of 3.3 cm × 4.7 cm, which is comparable to the 3.4 cm × 3.4 cm resolution reported for the MSBP benchmark while using a substantially smaller array than the 200 × 400 configuration adopted in that benchmark. The processing time of the proposed method is 0.023 s, which is significantly lower than the 1420.29 s reported for MSBP, demonstrating the computational efficiency of Fourier-based spectral processing. In comparison, although the multipath-based method has lower computational complexity, its resolution is worse than 20 cm and is therefore insufficient for centimeter-scale discrimination of threat-object contours in security-screening applications. In addition, point-target simulations show that the proposed block-segmentation method extends the effective imaging area from a local region of approximately 0.2 m × 0.2 m to the full 1 m × 1 m imaging plane, corresponding to a fivefold increase in linear imaging span and a 25-fold increase in imaging area. Therefore, the advantage of the proposed method does not lie in a single performance metric. Instead, through the joint design of block-segmented spatial-spectrum processing and microwave-photonic broadband reception, it simultaneously achieves centimeter-level resolution, extended near-field imaging coverage, and high computational efficiency with a relatively small array, providing a feasible technical solution for broadband, high-resolution millimeter-wave security-screening imaging.

This study primarily provides proof-of-principle validation of the proposed method; therefore, the experiments use aluminum-foil targets against a controlled background. In practical screening scenarios, strong reflections from the human body may obscure weakly scattering parts of the target, while multipath propagation between the body and the target may introduce additional clutter. The coherent superposition of this clutter and the target echoes can cause the measured phase to deviate from the ideal target-echo phase, distorting the spatial-spectrum matrices constructed for individual subblocks and consequently producing image distortion and artifacts.

## 6. Conclusions

This paper proposes a block-segmented spatial-spectrum imaging method for near-field millimeter-wave imaging and validates it using a Ka-band microwave-photonic broadband reception prototype. The method divides a large imaging region into multiple subblocks and establishes an independent local coordinate system for each subblock, thereby reducing the spatial offset of scatterers relative to the local coordinate origin. This suppresses high-order nonlinear phase errors, restores local linear spatial-frequency mapping, and enables large-area near-field imaging through sub-image stitching. Simulations with point targets and an aircraft-shaped complex target verify the basic mechanism of the proposed method and its capability for complete reconstruction of large-area near-field targets. A 32–38 GHz Ka-band near-field imaging system is further developed and experimentally evaluated in combination with a microwave-photonic broadband receiver. The resolution experiment achieves a two-dimensional imaging resolution of approximately 3.3 cm × 4.7 cm, confirming that broadband spectral resources improve spatial-spectrum imaging resolution by expanding the two-dimensional spatial-frequency support. The gun-shaped target experiment further demonstrates that block-segmented processing extends the effective imaging region from approximately 0.2 m × 0.2 m to 1 m × 1 m, corresponding to a fivefold increase in linear imaging span and a 25-fold increase in imaging area. Overall, the joint design of block-segmented spatial-spectrum processing and microwave-photonic broadband reception enables large-area, high-resolution, and computationally efficient imaging under a limited physical aperture and short-range near-field conditions, providing a feasible technical route for wide-field near-field applications such as millimeter-wave security screening. Future work will extend the method to three-dimensional spatial-spectrum millimeter-wave imaging, investigate adaptive subblock partitioning and local coordinate optimization for curved human-body surfaces and complex target geometries, and incorporate high-speed parallel microwave-photonic processing to advance millimeter-wave security screening from two-dimensional static imaging toward three-dimensional, dynamic, and real-time operation.

Future work will include body-phantom or human-body-equivalent experiments with clutter suppression to evaluate imaging performance under more realistic screening conditions. Building on this foundation, we will conduct further research into methods for identifying real threats and simulators that closely resemble them, with the aim of advancing the application from proof-of-concept to practical use in personnel security screening.

## Figures and Tables

**Table 1:** Quantitative comparison of imaging methods.

Imaging Method	Proposed Method	MSBP Benchmark [16]	Multipath-Based Method [17]
Array configuration	3 × 13	3 × 13	1 × 1
Resolution (cm)	3.3 × 4.7	3.4 × 3.4	>20
Processing time (s)	0.023	1420.29	0.001

## Data Availability

The data presented in this study are available from the corresponding author upon reasonable request.

## Abbreviations

The following abbreviations are used in this manuscript:

AWG	Arbitrary waveform generator
BP	Backprojection
EIRP	Equivalent isotropically radiated power
FFT	Fast Fourier transform
GSAFT	Generalized synthetic-aperture focusing technique
IF	Intermediate frequency
LFM	Linear frequency modulation
LNA	Low-noise amplifier
LO	Local oscillator
MIMO	Multiple-input multiple-output
mm-wave	Millimeter-wave
MZM	Mach–Zehnder modulator
OSC	Oscilloscope
PSLR	Peak sidelobe level ratio
RMA	Range migration algorithm
SNR	Signal-to-noise ratio
